# CNTs-Modified Nb_3_O_7_F Hybrid Nanocrystal towards Faster Carrier Migration, Lower Bandgap and Higher Photocatalytic Activity

**DOI:** 10.1038/srep39973

**Published:** 2017-01-06

**Authors:** Fei Huang, Zhen Li, Aihua Yan, Hui Zhao, Huagen Liang, Qingyu Gao, Yinghuai Qiang

**Affiliations:** 1 Low Carbon Energy Institute, China University of Mining & Technology, Xuzhou 221008, China; 2 School of Materials Science and Engineering, China University of Mining & Technology, Xuzhou 221116, China; 3 School of Chemical Engineering & Technology, China University of Mining & Technology, Xuzhou 221116, China.

## Abstract

Novel semiconductor photocatalysts have been the research focus and received much attention in recent years. The key issues for novel semiconductor photocatalysts are to effectively harvest solar energy and enhance the separation efficiency of the electron-hole pairs. In this work, novel Nb_3_O_7_F/CNTs hybrid nanocomposites with enhanced photocatalytic activity have been successfully synthesized by a facile hydrothermal *plus* etching technique. The important finding is that appropriate pH values lead to the formation of Nb_3_O_7_F nanocrystal directly. A general strategy to introdue interaction between Nb_3_O_7_F and CNTs markedly enhances the photocatalytic activity of Nb_3_O_7_F. Comparatively, Nb_3_O_7_F/CNTs nanocomposites exhibit higher photodegradation efficiency and faster photodegradation rate in the solution of methylene blue (MB) under visible-light irradiation. The higher photocatalytic activity may be attributed to more exposed active sites, higher carrier migration and narrower bandgap because of good synergistic effect. The results here may inspire more engineering, new design and facile fabrication of novel photocatalysts with highly photocatalytic activity.

As a new family of semiconductor photocatalysts, niobium oxyfluoride (Nb_3_O_7_F, labeled as NOF) nanomaterials with TiO_2_-similar electronic structure and energy band structure have recently attracted wide attention because they exhibit excellent photocatalytic activities due to the advantages of efficient light absorption, low carrier recombination and stable phase^1^,^2^,^3^. However, the practical application still suffers from the low photocatalytic efficiency in sewage disposal because of their intrinsic characteristics. A common explanation includes their large bandgap with 3.12 eV and relatively low quantum efficiency^4^,^5^. It is well known that the photon absorption depends strongly on their bandgap energy, dispersibility, crystal structure, *etc.* Usually, the photons can only be absorbed by the photocatalyst if the photon energy is higher than the bandgap energy^6^,^7^. Therefore, it is imperative to develop some new methods to narrow the bandgap of NOF photocatalysts or to widen band edge absorption threshold towards visible light, to decrease the high carrer recombination rate and to enhance the carrier separation efficiency.

Recently, the most-successful strategy for hybrid technique and multicomponent heterojunctions (Schottky barrier) has caused extensive concern to solve the same problems confronting TiO_2_ photocatalysts^8^,^9^,^10^,^11^,^12^. This method can markedly improve the carrier separation, quantum efficiency and photocatalytic activities, through enhancing the extraction of photoexcited electrons and suppressing charge carrier recombination probability^13^. Moreover, some new features and synergistic effects can usually induce some unexpected results, especially bandgap adjustment, carrier lifetime, *etc*. Particularly, carbon nanotubes (CNTs) have been the scientific focus of photocatalytic application due to their high surface area and good electron mobility^14^,^15^,^16^. However, the majority of reported CNTs/TiO_2_ nanocomposites still suffer from low photodegradation effeciency due to the aggregates and weak interfacial control in the pratical application. Usually, an ideal interface between catalysts and CNTs should combine efficient electron transport and the possibility to improve its optical response^17^,^18^. However, the development of new heterostructures and controllable preparation method still remains a significant challenge for scientific community. Moreover, the photocatalytic performance of NOF/CNTs hybrid photocatalysts was still largely unexplored.

In the present work, we employ a hydrothermal *plus* etching method to prepare NOF/CNTs nanocomposites with enhancing photocatalytic activities. To the best of our knowledge, it is the first systematical report of the successful preparation of NOF/CNTs hybrid nanocomposites. Given that CNTs tend to aggregate during the preparation process, we pre-treated CNTs in acidic solution. The interaction between NOF and CNTs results in more active sites and rapider diffusion of photogenerated carriers, as well as narrower bandgap. More importantly, the photocatalytic activities of as-synthesized NOF/CNTs nanocomposites are geatly enhanced in the photodegradation of MB solution. This finding may be of interest to materials scientists and the method may offer new inspirations to synthesize and design some novel photocatalysts with high activity.

## Results and Discussion

### Synthesis and Structural Characterization of NOF Nanomaterials

In order to solve the traditonal problems of poor compositional homogeneity and large particle size, here we adopted solvothermal *plus* etching method to synthesize NOF nanomaterials. We have initially noticed that chemical etching method using HF as etching solution is an effective route^5^,^6^. Subsequently, Zhang also prepared NOF nanomaterials using a hydrothermal method, but three key issues should be further clarified^4^. Firstly, the annealing at 550 °C would damage NOF crystal configuration, whatever in an oxygen-free atmosphere or in an oxygen-containing atmosphere. NOF would rapidly decompose above 500 °C, as shown in Figures S1 and S2. Secondly, the growth mechanism based on the reaction of Nb_2_O_5_ with HF need to be further confirmed (See Figure S3). Thirdly, the importance of HF etching solution should be further stressed.

**Figure 1:**
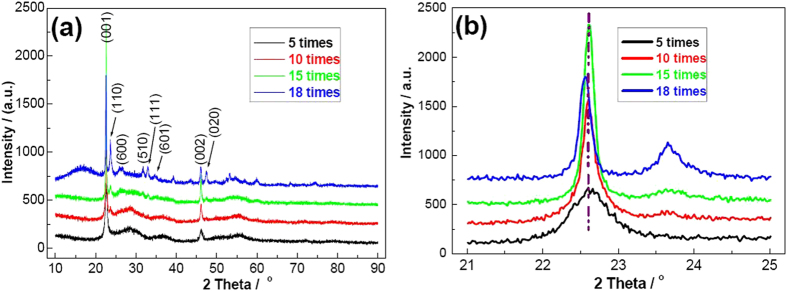
XRD patterns of the samples prepared with different HF concentrations. (**a**) Wide-scan; (**b**) local high-resolution scan.

Here we reveal for the first time that appropriate concentration of HF is an important factor for controlling the crystalline phases of NOF. In order to clarify the critical reaction, HF/NbCl_5_ molar ratio varied from 5 to 25 times by keeping other parameters fixed to understand growth behavior. The effect of HF on the final crystallite phases was examined using XRD equipment in detail. As shown in Figure 1a, all the samples demonstrate high crystalline nature. When the molar ratio is 5 times and 10 times, the products tend to form NOF. However, some typical peaks of NOF don’t appear in XRD patterns, especially (110) and (020). Further increasing the concentration to 15 times, it is very interesting that all the diffraction peaks can be indexed as typical orthorhombic NOF with parameters of *a* (Å) = 20.67, *b* (Å) = 3.833, *c* (Å) = 3.927 (JCPDS No. 18–0915). The main peaks at 2θ = 22.6°, 23.5°, 25.8°, 31.7°, 32.9°, 34.5°, 46.1° and 47.8°, correspond to (001), (110), (600), (510), (111), (601), (002) and (020) crystalline planes, respectively. No other peaks can be observed, indicating that the samples are made up of NOF. It should be stressed that the relative intensities of (001)/(100) planes vary distinctly from the standard data, implying that the preferential growth direction of NOF is the [001] direction. When the concentration increases to 18 times, some other unknown peaks present in XRD profiles. The possible cause is that some other niobium oxyfluorides with high-content fuoride are present in the products. Moreover, the peak of (001) obviously shifts to a small angle (Fig. 1b). We also try to further increase HF concnetration to 25 times. Unfortunately, it is hard to collect precipitates, indicating that NOF can be dissolved under higher concentration of HF. In short, appropriate pH value is an important factor for synthesize NOF nanomaterials. This is the main cause that the addition of HCl could also contribtue to the formation of NOF^19^.

**Figure 2:**
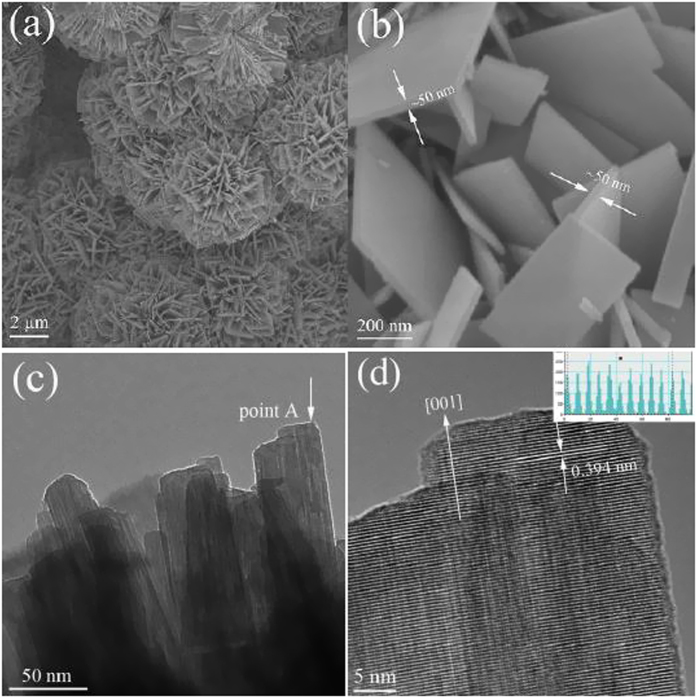
FESEM and HRTEM images of the samples synthesized at 180 °C for 24 h. (**a**), (**b**) FESEM images; (**c**), (**d**) HRTEM images.

FESEM and HRTEM results confirm that reaction time could markedly change the morphological evolution and the final products are evolved from nanoparticles, nanosheets, nanobricks (See Figure S4). The typical products prepared at 180 °C for 24 h are dominated by nanowall structure with 8–10 μm in diameter (Fig. 2a). The magnified image shows that the nanostructures are independent nanosheets with about 50 nm in thickness, about 1 μm in length and 200–300 nm in width (Fig. 2b). The high resolution TEM image for the edge of NOF nanostructure (Point A in Fig. 2c), as displayed in Fig. 2d, reveals that NOF nanosheets with single-crystalline nature have been synthesized simply by a facile hydrothermal *plus* etching approach. The distance between the lattice planes along nanosheet growth is 0.394 nm, which corresponds to (001) d-space of NOF. The result implies that the preferential growth or the periodic growth of the nanosheet is along [001] direction, further confirming the judgement of above-mentioned XRD results.

### Effect of CNTs Modification on the Strucutrue and Absorption Spectrum of NOF Nanomaterials

**Figure 3:**
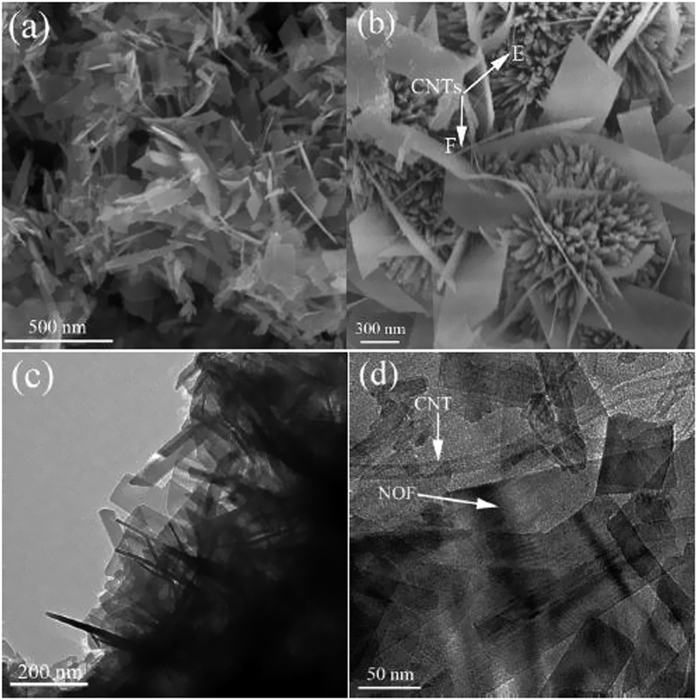
Typical SEM and TEM images of NOF-0.5CNTs sample.

**Figure 4:**
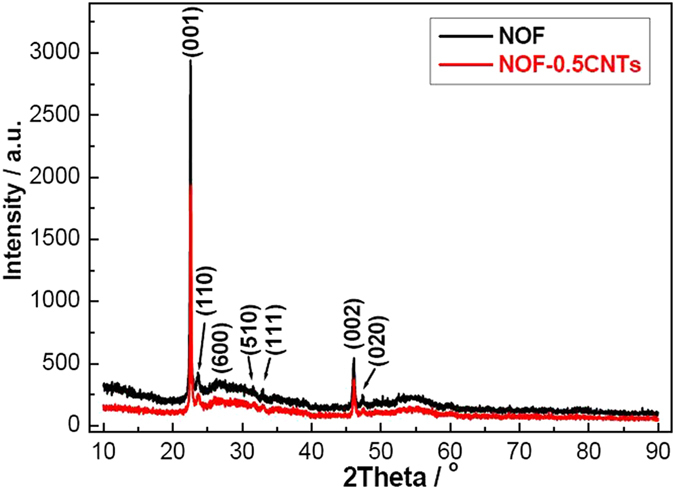
XRD patterns of NOF and NOF-0.5CNTs samples.

To explore novel phenomenon and confirm the interaction between NOF and CNTs, here SEM and TEM techniques were used to characterize NOF/CNTs samples with different CNTs contents (Fig. 3). It should be noted that the hydrothermal time is shortened to 12 h in order to find out the possible intermediate process and microstructure changes. As shown in Fig. 3a and c, the samples keep unchanged after addition of CNTs. The magnified SEM image shows that there are two possible states for CNTs, namely, dispersion on the NOF surface and heterojunction with NOF, as shown by E point and F point in Fig. 3b. TEM image further confirms that CNTs are fixed on NOF nanosheets with good dispersion (Fig. 3d). XRD results show that the phase keeps unchanged after adding a small amount of CNTs (Fig. 4). However, the intensity of NOF-0.5CNTs sample decreases rapidly, compared with that of pure NOF. The possible cause is that the crystallinity of CNTs is much lower than that of NOF.

**Figure 5:**
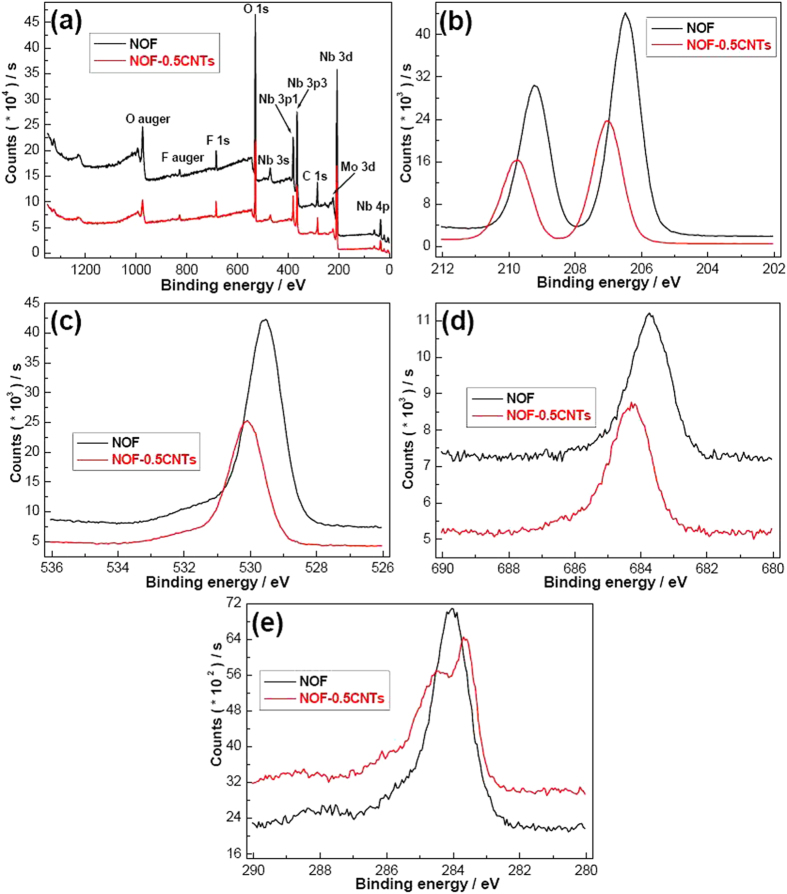
XPS spectra of NOF and NOF-0.5CNTs samples. (**a**) Wide scan; (**b**) narrow scan of Nb3d; (**c**) narrow scan of O1s; (**d**) narrow scan of F1s; (**e**) narrow scan of C1s.

To further confirm the slight changes of the structure, chemical states and composition, XPS was used to further characterize the samples before and after addition of CNTs. As shown in Fig. 5, both NOF and NOF-0.5CNTs contain Nb, O and F elements, demonstrating that the final products are NOF (Fig. 5a). The narrow scan of Nb element indicates that NOF has two obvious binding energies of 206.49 and 209.24 eV, corresponding to Nb 3d_3/2_ and Nb 3d_5/2_, respectively (Fig. 5b). The two peaks could be ascribed to Nb–F chemical bonding^20^. The peaks of NOF at around 529.54 and 683.74 eV can be ascribed to O1s corresponding to typical Nb-O band and F1s corresponding to typical Nb-F band (Fig. 5c,d)^16^,^21^. Importantly, all the peaks of Nb3d, O1s and F1s for NOF/CNTs samples shift to higher binding energy, implying that some potential interactions present in the final products. The weakened peak intensity of Nb3d, O1s and F1s may be caused by partially amorphous form of CNTs. The peaks at about 284.04 eV for NOF samples can be ascribed to sp^3^ carbon, which results from the testing method and the sampling method (Fig. 5e). However, the narrow scan for NOF-0.5CNTs sample presents three peaks at 283.09, 284.49 and 288.9 eV. The spliting peaks at 283.09 and 284.49 eV can be ascribed to the C-Nb band and C-O band, further implying that there is a strong interaction between NOF and CNTs^22^,^23^. The shoulder peak at about 288.9 eV corresponds to C–F bonding configuration^24^. In a word, the co-electron cloudy between Nb atom and C atom, or C atom and F atom, may result in higher electron concentration, which would be helpful to the seperation of photogenerated carriers.

**Figure 6:**
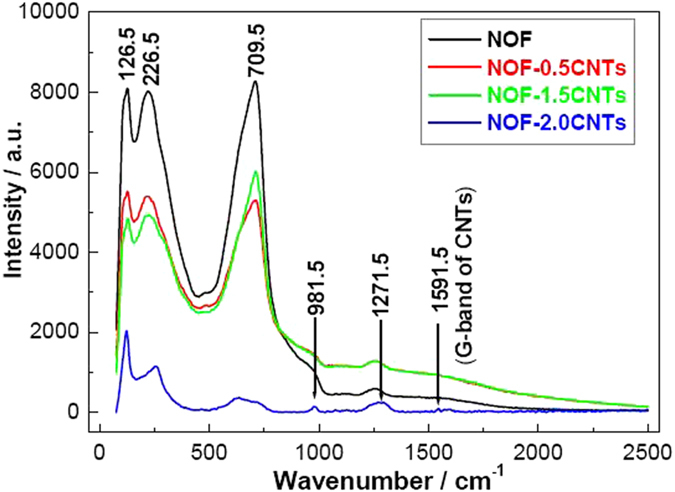
Raman spectra of NOF and NOF/CNTs nanocomposites.

Additionally, Raman spectroscopy is a kind of powerful tool to characterize CNTs-based materials. In this work, Raman studies were performed on pure NOF and NOF/CNTs nanocomposites, as shown in Fig. 6. The results indicate that both NOF and NOF/CNTs exhibit typical Nb–F vibrational band^2^, O = Nb = O twisting band^25^ and Nb–O–Nb stretching vibration band^26^ at 126.5 cm^−1^, 226.5 cm^−1^ and 709.5 cm^−1^. Compared with NOF, all the intensities of typical peaks obviously decrease after addition of CNTs. Importantly, there is a new band at 1591.5 cm^−1^ when over 0.5 wt.% CNTs is added. The peak can be attributed to typical G-band of CNTs due to vibration of carbon atoms along the axis, indicating that ordered graphite CNTs present in the sample^27^. Moreover, the peak becomes stronger with the increase of CNTs. Another important characteristic is that the peak of D-band of CNTs in the range of 1310–1350 cm^−1^ can not be observed for NOF/CNTs sample^28^. However, there is an evident difference between pure NOF and NOF/CNTs sample at about 1271.5 cm^−1^. The cause may result from the synergistic effect between O-Nb-O stretching mode at 1261.5 cm^−1^ and D-band of CNTs. Consequenly, the peak gradually shifts to 1271.5 cm^−1^. An obvious peak at 981.5 cm^−1^ is also observed and can be associated with slight distortion Nb=O asmmetric stretching mode when CNTs arrive at 2.0 wt.%, implying the synergistic effect between NOF and CNTs^29^,^30^,^31^. Therefore, it is reasonalbly deduced that a strong interaction between NOF and CNTs, namely the formation of heterostructure, results in the new Nb-C band because the hydrophilic pretreatment of CNTs results in the reaction between CNTs and the containing-Nb intermediate products, which is in well agreement with SEM images and XPS results.

**Figure 7:**
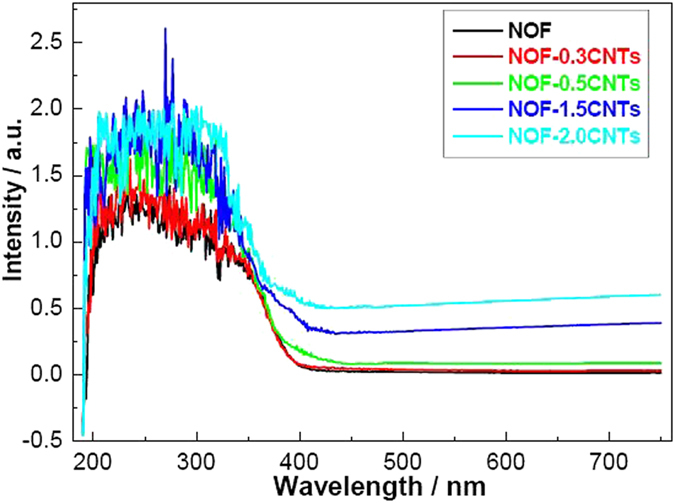
UV-vis spectra of NOF and NOF/CNTs nanocomposites.

To investigate the influence of CNTs modification on the optical absorption of NOF, UV-vis spectra were also characterized in this work. As shown in Fig. 7, NOF materials have a strong absorption in the UV-vis light range and show the characteristic absorption sharp edge at around 410 nm. Moreover, there is almost no difference between NOF and NOF-0.3CNTs. However, as expected, it could be observed that NOF-0.5CNTs, NOF-1.5CNTs and NOF-2.0CNTs show higher optical absorption edge for wavelengths than pure NOF. An obvious red shift of about 25 nm is observed. It is noticeable that the introduction of CNTs leads to an increase of optical absorption in the visible-light range, indicating a decrease in the bandgap energy for NOF/CNTs nanocomposite. The increase of optical absorption may be attributed to the creation of an electronic interphase interaction between CNTs and NOF. According to band theory, the bandgaps (*E*_*g*_) of the NOF, NOF-0.5CNTs, NOF-1.5CNTs and NOF-2.0CNTs are calculated to be 3.09, 2.95, 3.02, 2.99 eV, respectively. Generally speaking, CNTs as a photosensitizer do not change band gap except impoving carrier migration and active sites^32^. A plausible explanation is that the formation of heterostructure with new carbon-oxygen-niobium bond contributes to the interphase interaction, which further demonstrates above-mentioned results^33^,^34^.

### Grwoth Mechanism of NOF/CNTs Hierarchitectures

To understand the role of CNTs in NOF nanowall, here we proposed the possible chemical reactions according to above results and previous works^3^,^4^. Firstly, NbCl_5_ is etched in HF solution and forms H_2_NbF_7_ intermediate states (Equation 1, 2, 3). Subsequently, NbF_7_^2-^ further forms NOF through hydrolyzation reaction (Equation 4). In a word, NbCl_5_ can react with HF in the aqueous solution and finally form NOF.


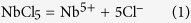



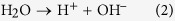



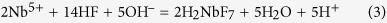



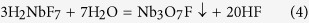


**Figure 8:**
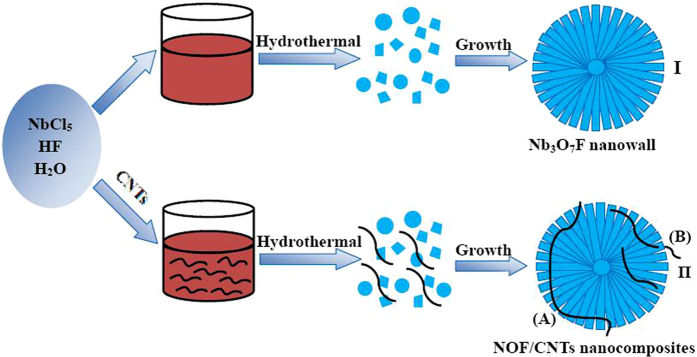
Schematic illustration for the synthesis of NOF and NOF/CNTs nanocomposites.

Therefore, the growth mechanism of NOF/CNTs nanomaterials can be explained as following three steps. Initially, niobium source forms soluble NbF_7_^2−^ ions under strong acid condition. The second step is that NbF_7_^2−^ ions undgo hydrolyzation reaction and form NOF precipitation. The last step is that NOF precipitations attach each other and further grow into one-dimentional nanoneedles and two-dimentional nanosheets, which can be confirmed by SEM images in Figure S4. The driving force of forming nanosheets with primary NOF nanoparticles and nanoneedles is to reduce the total surface energy of NOF crystals during Oswald ripening process. Continuously, NOF nanosheets grow out from the core and form nanosheets morphology, which finally leads to the formation of nanowall structure. It should be stressed that CNTs doesn’t take participate in the chemical reaction during the growth process. But H-bond of CNTs with acidic treatment will contribute to the bonding interaction with H_2_NbF_7_ intermediate state. Consequently, the heterojunction would be present after hydrolyzation reaction. The growth mechanism and synthesis procedure of NOF/CNTs nanomaterials are illustrated in Fig. 8.

### Photocatalytic Activity and Mechanism Discussion of NOF/CNTs Catalysts

**Figure 9:**
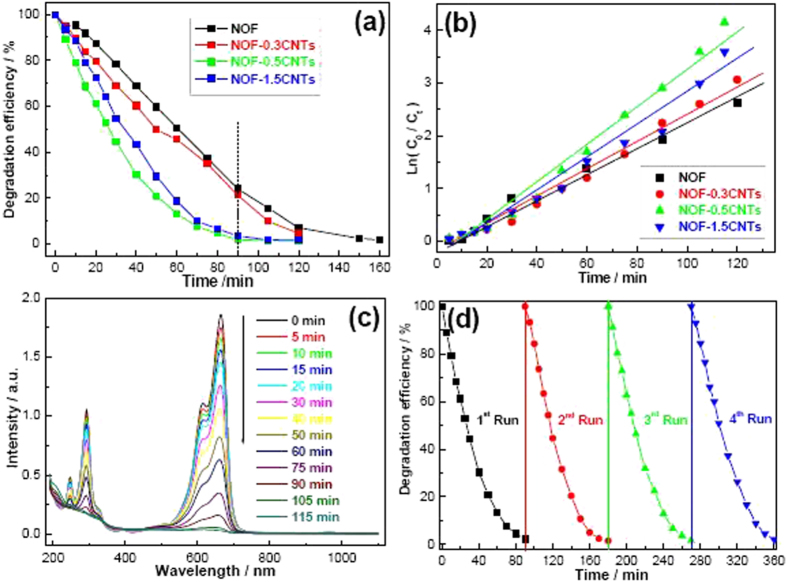
Photodegradation curves of MB aqueous solution for different samples. (**a**) Photodegradation *versus* time curves; (**b**) kinetic rate curves; (**c**) UV-vis absorption spectral curves as a function of irradiation time in the presence of NOF-0.5CNTs; (**d**) cycling curves of photodegradation efficiency for NOF-0.5CNTs photocatalysts.

To demonstrate the effectiveness and quantify the photocatalytic activity of NOF/CNTs nanocomposites, the photocatalytic activity of NOF/CNTs nanocomposites was evaluated by monitoring the degradation of MB aqueous solution under visible-light irradiation. As shown in Fig. 9a, all the samples can achieve their final photodegradation efficiency after 180, 120, 90 and 105 min for NOF, NOF-0.3CNTs, NOF-0.5CNTs and NOF-1.5CNTs samples, respectively. That is, the photodegradation rate is markedly improved after the addition of CNTs. Moreover, NOF/CNTs samples have better photocatalytic activity than bare NOF from the kinetic behaviors. About 79.7%, 98.1% and 96.5% of MB are degraded after 90 min for NOF-0.3CNTs, NOF-0.5CNTs and NOF-1.5CNTs samples. While pure NOF samples only exhibit 75.9% decolorization rate in the same time.

Figure 9b displays the curve of logarithmic form in order to specify the first order nature of the reaction and facilitate the calculation of first-order reaction rate constants (*k*)^1^,^35^. Namely, the photodegradation reaction approximately obeys the linear relationship between *ln(C*_*0*_*/C)* and irradiation time, which is expressed as follows:


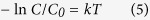


where *T* (min) is the irradiation time and *k* (min^−1^) denotes the overall rate constant. According to the Equation 5, the *k* value for each catalyst is obtained from the slopes of the lines of best fit, as listed in Table 1. The calculated *k* values are 0.140, 0.232, 0.322 and 0.311 min^−1^ for NOF, NOF-0.3CNTs, NOF-0.5CNTs and NOF-1.5CNTs samples, respectively. Specifically, NOF-0.5CNTs shows the highest *k* value, further indicating that NOF/CNTs catalysts exhibit higher photocatalytic performance than that of bare NOF catalyst. Figure 9c demonstrates rapid decrease of the main peak intensity of MB in the presence of NOF-0.5CNTs catalysts. In another word, the visible-light activity of NOF is enhanced after addition of CNTs. The cycle experiments show that the photodegradation efficiency in 90 min tends to remain stable at about 97.7%, demonstrating that NOF/CNTs photocatalysts have good stability (Fig. 9d).

The basic process might be attributed to the following process: Under visible-light irradiation, photo-excited electrons are injected into the conduction band of NOF and reduce the surface adsorbed O_2_ to form O_2_^•−^ (Equation 6); then O_2_^•−^and *h*^+^ can reace with H_2_O to form OH^•^ radicals through a series of reactions (Equation 7, 8, 9); finally, dye molecules are degraded by the OH^•^ radicals or H^+^ radicals (Equation 10 and 11)^36^,^37^.


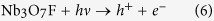



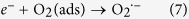



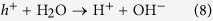



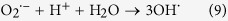



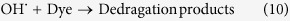



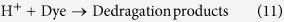


**Figure 10:**
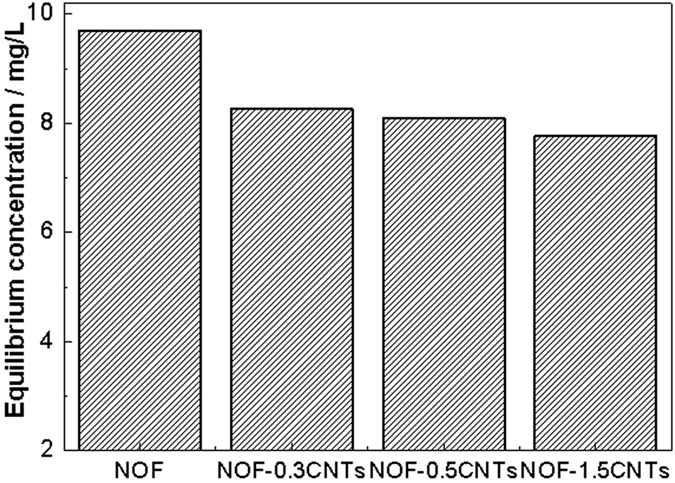
Adsorption equilibrium concentration of MB for different photocatalysts.

**Figure 11:**
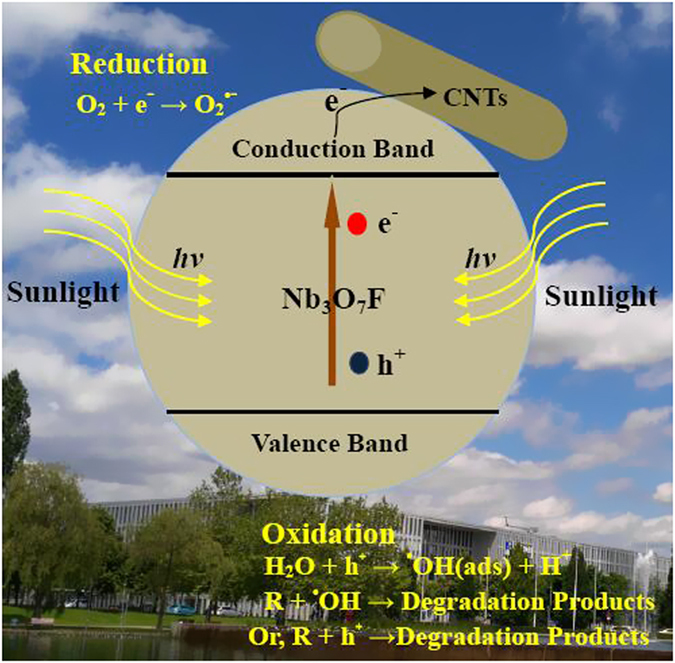
Schematic illustration of the photocatalytic mechanism of as-synthesized NOF/CNTs photocatallyst.

Therefore, the enhanced photocatalytic performance can be explained as following causes: Firstly, CNTs as a carrier can adsorb more dye molecules and markedly expose more active sites of NOF catalysts, which can provide much more photodegradation probability^38^,^39^. As shown in Fig. 10, it can be clearly observed that the adsorption equilibrium concentration clearly decreases with the increase of CNTs contents, indicating that the adsorption capacity increases greatly after addition of CNTs. Secondly, CNTs acting as photosensitizer can obviously improve the transport and carrier migration, as well as reduce recombination activity of e^−^/h^+^ pair^40^. In the presence of CNTs, the photogenerated electrons can freely move towards CNTs surface because of perfect heterostructure contact, causing excessive valence band holes within NOF to migrate to its surface. Consequently, charge transfer to the adsorbed species is continuous. Thirdly, the excited CNTs can inject a single electron into the separated hole of NOF and favor separation of the photogenerated charge carriers through formation of the Schottky barrier at NOF/CNTs interface, which is confirmed by UV-vis absorption spectra. In another word, the heterostructure with a lower Fermi level slightly changes the bandgap of NOF after CNTs are introduced^16^,^41^. That is to say, the efficiency for bare NOF catalysts is improved because of more exposed active sites, higher interfacial electron migration rate and narrower wide bandgap. The photocatalysis mechanism is shown in Fig. 11.

## Conclusions

In summary, NOF/CNTs hybrid nanocomposites as a kind of novel photocatalyst have been successfully synthesized *via* a facile hydrothermal *plus* etching technique in present work. In this architecture, the semiconductor NOF acts as photocatalysts to capture solar energy while CNTs serve as co-catalyst to further promote the separation and transfer of photogenerated carriers, as well as visible-light absorption. The experimental and calculated results show that NOF/CNTs nanocomposites have higher photodegradation efficiency and faster photocatalytic rate, compared to bare NOF materials. Analyzing combinatorial architecture and photocatalytic performance in the nanocomposites provides a shortcut to understanding physical-functional property relationships in depth and offers a rapid method for discovering new materials and new photocatalysts.

## Experimental Section

### Raw Materials

CNTs (98%, 6–13 nm in outer diameter, 2.5–20 μm in length) and NbCl_5_ (99.95 wt.%) were purchased from Alfa Aesar Incorporation. HF (30 wt.%), nitric acid (HNO_3_, 65 wt.%) and MB were received from Sinopharm Chemical Reagent Co., Ltd. All chemicals were of analytical grade and used without further purification.

### Synthesis of NOF/CNTs nanomaterials

To improve the hydrophily, CNTs were pretreated in HNO_3_ solution. In a typical synthesis process, 0.5 g of CNTs was mixed with 30 mL HNO_3_ under ultrasonic dispersion for 10 min and then heated to 80 °C for 2 h under continuous stirring. The resulting solution was cooled to room temperature, filtered, washed and dried at 80 °C for 4 hours. Then, a small amount of dried CNTs was dispersed in 80 mL of water and sonicated for 1 h to obtain a uniform suspension. Subsequently, NbCl_5_ (0.006 mol) and HF (0.03 mol) were added into above suspension. And then the mixed solution was transferred into a 100 mL teflon-lined autoclave, sealed, maintained at 180 °C for 24 h and followed by natural cooling to room temperature. Afterwards, the products were centrifugated and washed with deionized water and anhydrous ethanol for several times, respectively. The final products were dried at 80 °C for 3 h. It should be stressed that the content of CNTs was calculated according to final amounts of NOF. For convenience, the samples with 0.3 wt.%, 0.5 wt.%, 1.0 wt.%, 1.5 wt.%, 2.0 wt.% and 3.0 wt.% CNTs were named as NOF-0.3CNTs, NOF-0.5CNTs, NOF-1.0CNTs, NOF-1.5CNTs and NOF-2.0CNTs, respectively.

### Materials Characterizations

X-ray diffraction (XRD, Bruker D8, German) with Cu *Ka* radiation was used to identify the crystallinity and phase of final products. The scan rate was 5 °/min in the 2θ range of 10–90°. X-ray photoelectron spectroscopy (XPS, Thermo Fisher Escalab 250XI, USA) with an Al *Ka* source was applied to characterize the chemical states of the samples. Field emission scanning electron microscopy (FESEM, Hitachi S4800, Japan) and high resolution transmission electron microscopy (HRTEM, FEI Tecnai G2 F20, USA) were selected to observe the particle size, surface microstructure and morphological evolution of NOF/CNTs catalysts. Raman spectra were recorded on a Renishaw-2000 Raman spectrometer using Ar ion laser excitation with a 532 nm wavelength excitation. UV–vis spectrophotometer (Analytik Jena Specord®210, German) with 75 mm integrating sphere was employed to analyze the optical absorption of NOF/CNTs catalysts.

### Photocatalytic activity evaluation

The photocatalytic activity of NOF and NOF/CNTs catalysts was evaluated by measuring the degradation of MB aqueous solution in a photoreactor (CEAULIOHT Ltd. Co., CEL-LB70, China) with a xenon lamp. The visible light with wavelengths of 380–780 nm comes from the xenon lamp treated under two cutoff filters (JB 380 and VISREF 350–780, China). Firstly, 20 mg of photocatalysts and 200 mL aqueous solution of MB (10 mg L^−1^) were mixed in quartz vessel. Then, the mixture was placed inside the photoreactor in which the vessel was 15 cm away from visible-light sources. Before irradiation, the mixture was magnetically stirred in dark for 1 h to achieve adsorption-desorption equilibrium. Thereafter, the suspension was irradiated at room temperature and 5 mL solution was collected from quartz vessel and centrifuged at regular intervals. The changes of MB concentration were analyzed using an UV-vis spectrometer and calculated according to the absorbance intensity at 665 nm. Before testing, blank experiments without catalyst were carried out. (C_0_-C)/C_0_ was used to describe the photodegradation efficiency, where C was the concentration after irradiation for a certain regular interval and C_0_ was the initial concentration after adsorption-desorption equilibrium in the dark.

## Additional Information

**How to cite this article**: Huang, F. *et al*. CNTs-Modified Nb_3_O_7_F Hybrid Nanocrystal towards Faster Carrier Migration, Lower Bandgap and Higher Photocatalytic Activity. *Sci. Rep.* **7**, 39973; doi: 10.1038/srep39973 (2017).

**Publisher's note:** Springer Nature remains neutral with regard to jurisdictional claims in published maps and institutional affiliations.

## Supplementary Material

Supplementary Information

## Figures and Tables

**Table 1:** Parameters and reaction kinetic equations for different samples.

Samples	Reaction kinetic equation	Rate constant (min^−1^)	R
NOF	*ln(C*_*0*_*/C)*=*0.140x-0.317*	0.140	0.926
NOF-0.3CNTs	*ln(C*_*0*_*/C)*=*0.232x-0.504*	0.232	0.947
NOF-0.5CNTs	*ln(C*_*0*_*/C)*=*0.322x-0.686*	0.322	0.959
NOF-1.5CNTs	*ln(C*_*0*_*/C)*=*0.239x-0.470*	0.239	0.968
