# Supplementary material for: CNTs-Modified Nb3O7F Hybrid Nanocrystal towards Faster Carrier Migration, Lower Bandgap and Higher Photocatalytic Activity
Source: Sci Rep. 2017 Jan 6;7:39973. doi: 10.1038/srep39973 (PMC5216398; doi:10.1038/srep39973)
Supplement: Supplementary Information [file srep39973-s1.doc]

**Supporting Information**

*Title: CNTs-Modified Nb_3_O_7_F Hybrid Nanocrystal towards Faster Carrier Migration, Lower Bandgap and Higher Photocatalytic Activity*

**Author(s):** Fei Huang*, Zhen Li, Aihua Yan*, Hui Zhao, Huagen Liang, Qingyu Gao and Yinghuai Qiang


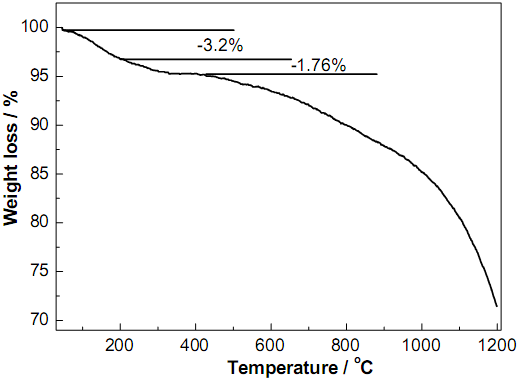


**Figure S1** TG/DSC curves of the NOF samples

From the TG curve (**Figure S1**), it can be seen that there are three obvious stages of weight loss at the temperature range of 25~1200 ^o^C. In the third stage, the product will continue to lose weight over 430 ^o^C, indicating that the thermal stabiliting is broken out over 430 ^o^C.

**Figure S2** shows the curves of the oxidation kinetics of NOF at 400 ^o^C, 500 ^o^C and 700 ^o^C. It can be seen that mass loss increases rapidly with the increasing of temperature and time, showing the importance of thermal staility of NOF materials. It also can be seen that Nb_3_O_7_F materials will continue decomposition over 500 ^o^C, which further confirm the correct result of **Figure S1**.


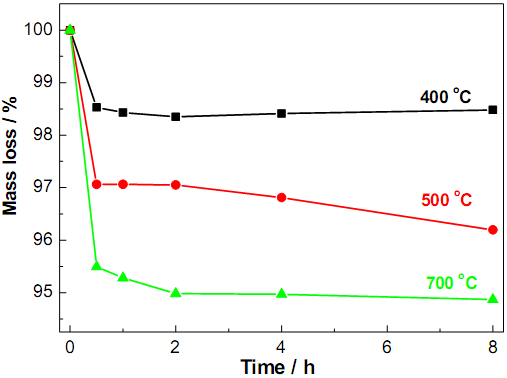


**Figure S2** Mass loss curves of NOF treated at different temperatures


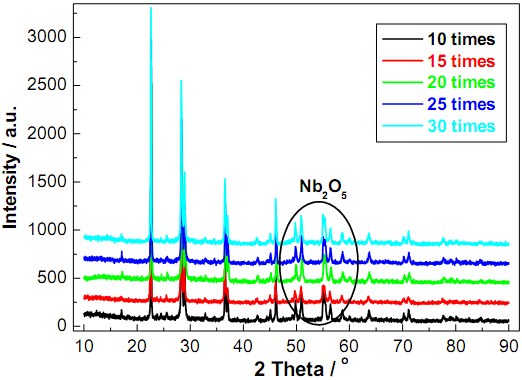


**Figure S3** XRD patterns of the samples prepared using N_2_O_5_ as raw materials

If the growth mechanism is based on the reaction of Nb_2_O_5_ with HF, then using Nb_2_O_5_ as raw materials in the same condition could also get NOF nanomaterals under the same condition. On contrary, the experiment results found that it is hard to get NOF nanomaterals using Nb_2_O_5_ as raw materials, indicating that the growth mechanism should be re-considered (**Figure S3**). Bohnke also confirmed the same reslults (*Mol. Cryst. Liq. Cryst.* **1998**, 311, 23).


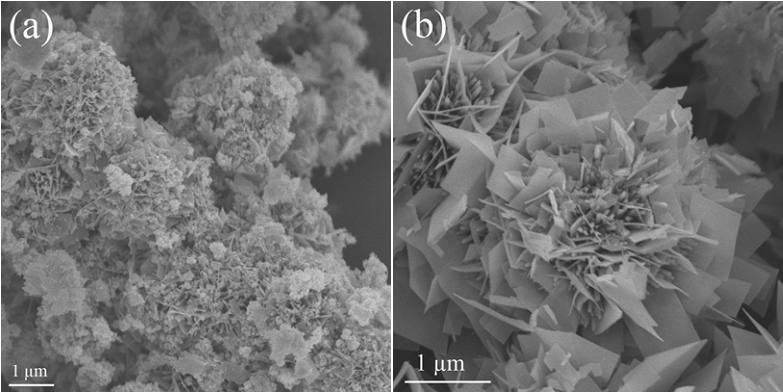


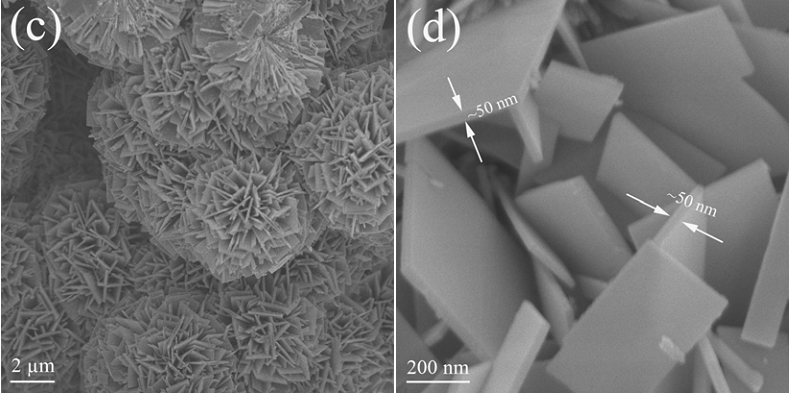


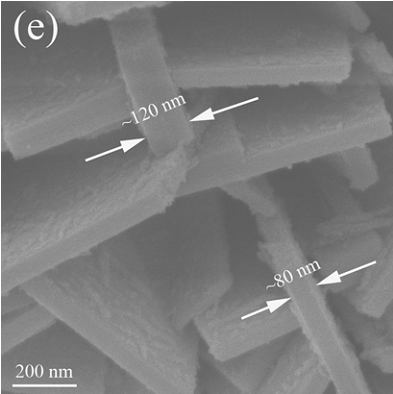


**Figure S4** Typical SEM images of the products prepared at 160 ^o^C for different reaction time. (a) 6 h; (b) 12 h; (c), (d) 24 h; (e) 48 h

In order to investigate the influence of reaction time on morphology evolution, the time-dependent experiment was conducted from 6 h to 48 h. The direct evidence of morphology evolution of Nb_3_O_7_F was demonstrated by SEM as shown in **Figure S4**. It is obviously found that reaction time could markedly influence the morphological evolution. In an early stage, irregular products can be ovserved when reaction proceeded for 6 h, including nanoparticle aggregations and nanosheets (**Figure S4a**). With the increase of reaction time to 12 h, there are less and less nanoparticles (**Figure S4b**). The nanoparticles are evolved to nanonods, implying that nanoparticle is a kind of intermediate states. The possible cause is that the nanoparticles attach together and further grow with the increase of reaction time. Further increasing reaction time to 24 h, the products are dominated by nanowall structure (**Figure S4c**). The magnified image shows that the nanostructures are independent nanosheets with about 50 nm in thickness, about 1 µm in length and 200-300 nm in width (**Figure S4d**). When the reaction time prolongs to 48 h, the morphology keeps unchanged. However, the nanosheet becomes obviously larger and the thickness increase to 100~120 nm (**Figure S4e**).
